# Hypomania Symptoms Across Psychiatric Disorders: Screening Use of the Hypomania Check-List 32 at Admission to an Outpatient Psychiatry Clinic

**DOI:** 10.3389/fpsyt.2018.00527

**Published:** 2018-11-07

**Authors:** Marta Camacho, Sílvia Almeida, Ana Rita Moura, Ana B. Fernandes, Gabriela Ribeiro, Joaquim Alves da Silva, J. Bernardo Barahona-Corrêa, Albino J. Oliveira-Maia

**Affiliations:** ^1^Champalimaud Clinical Centre, Champalimaud Centre for the Unknown, Lisbon, Portugal; ^2^Champalimaud Research, Champalimaud Centre for the Unknown, Lisbon, Portugal; ^3^Department of Psychiatry and Mental Health, Centro Hospitalar de Lisboa Ocidental, Lisbon, Portugal; ^4^NOVA Medical School/Faculdade de Ciências Médicas, Universidade Nova de Lisboa, Lisbon, Portugal; ^5^Lisbon Academic Medical Center PhD Program, Faculdade de Medicina, Universidade de Lisboa, Lisbon, Portugal

**Keywords:** hypomania, bipolar spectrum disorders, HCL-32, adaptation, European Portuguese

## Abstract

**Introduction:** Hypomania symptoms are best described as a continuum, ranging beyond Bipolar Spectrum Disorders (BSD). Other nosological entities, such as major depressive disorder, schizoaffective disorder, or borderline personality disorder, may also share symptoms with BSD, raising challenges for differential diagnosis. While the Hypomania Checklist-32 is one of the most widely used tools for screening hypomania, there is limited evidence describing its use in a real-world outpatient psychiatric clinical setting.

**Methods:** Here we tested the psychometric properties of a European Portuguese adaptation of the HCL-32, establishing its factor structure, reliability and construct validity. Furthermore, we analyzed differences in hypomanic symptoms among several clinical groups and in a non-clinical sample. Data was obtained retrospectively in an ecological setting from a clinical sample of an outpatient psychiatry and psychology clinic, comprising 463 Portuguese individuals, 326 of whom had a psychiatric diagnosis, namely BSD (*n* = 66), major depressive disorder (*n* = 116), or other psychiatric disorders (*n* = 144). A separate non-clinical sample was also collected among healthy volunteers (*n* = 62). A battery of self-report measures of affective symptoms was applied, and in a subset of patients, diagnosis was established using a structured diagnostic interview.

**Results:** Psychometric properties of the HCL-32 were adequate, with good internal consistency (Cronbach's α = 0.86) and test-retest stability (ICC = 0.86), and two subscores (“active/elated” and “risk-taking/irritable”) defined by Principal Component Analysis. Receiver Operating Characteristic curve analysis demonstrated that the test score discriminated moderately between patients with BSD and other clinical samples as well as healthy volunteers, with a cut-off score of 17 for the total score of the HCL-32 rendering the best combination of sensitivity and specificity. When compared to the HCL-32 total score, the risk-taking/irritable subscore seems to provide additional benefit in discriminating between different clinical groups, namely regarding specificity in the discrimination from patients with a diagnosis of major depressive disorder that was low for the full scale and the alternate subscale.

**Conclusions:** HCL-32 can be used as a screening tool for BSD among adult patients presenting in an outpatient psychiatric clinical setting.

## Introduction

The World Health Organization (1) estimates that 2.4% of people worldwide may suffer from Bipolar Spectrum Disorders (BSD), which have been proposed as one of the leading causes of years lost due to disability (2). In Portugal, where this study was conducted, the National Health Observatory, estimated a prevalence of medically confirmed Bipolar Disorder of 0.4% (3), highlighting the problems with unrecognized and misdiagnosed BSD in clinical practice. It is widely accepted that Bipolar Disorder, rather than a categorical condition, is best conceived as a continuum of disorders, varying in severity as well as other characteristics. The continuum ranges from bipolar disorder types I (BD-I) and II (BD-II), to cyclothymia and bipolar disorder not otherwise specified (2), that are clustered as BSDs. Hypomanic episodes are distinct periods of 4 or more days, with elevated, expansive or irritable mood, among other symptoms, that are observable by others (4), but of insufficient severity or compromise of functionality to meet criteria for full-fledged mania episodes (5, 6). While mania is more distinctive and easier to identify than hypomania, manic episodes are significantly less frequent than hypomania, and occur only in a specific subtype of BSD (BD-I) (4, 7). Thus, accurately identifying a current or prior episode of hypomania is decisive for the differential diagnosis of BSD.

Identifying a past history of hypomania can be difficult (8) and, as a result, BSDs are frequently misdiagnosed as unipolar major depressive disorder (5), borderline personality disorder (9), or other disorders. Consequences of such misdiagnosis include inadequate treatment and worsening of the disorder, inappropriate use of antidepressants, litigation and increased risk of suicide (10). To address these difficulties, several psychometric instruments have been developed to screen for hypomanic episodes and assess their severity. The Hypomania Checklist-32 (11) (HCL-32) is one of such self-report questionnaires, designed to screen for hypomania symptoms in patients with major depressive disorder. It is currently available in many languages and has been extensively studied (12–27). While the HCL-32 does not provide a formal diagnosis of BSD, it has been proposed as a valuable screening tool (28), with adequate psychometric properties, allowing for the assessment of BSD symptoms in an integral and standardized fashion. However, there is limited research exploring use of this instrument as a screening tool in an ecological context. One study, using the Italian version of the scale, tested the performance of the HCL-32 as a screening tool in a naturalistic psychiatric outpatient setting, demonstrating good screening accuracy of the scale, albeit in a relatively small sample of BSD patients (20). In more recent work conducted in a larger sample from both outpatient and inpatient settings in Korea, full and shortened versions of the scale had a similar screening performance to that described in previous work, but analyses were restricted to discrimination between BSD and major depressive disorder (29). Here, we focused on the profiles of hypomania symptoms in patients with BSD, when compared with patients with other psychiatric diagnoses, using the HCL-32 as a screening tool at admission to an outpatient psychiatry clinic. Specifically, after analyzing psychometric properties of a European Portuguese version of the HCL-32, including internal consistency, factor structure, test-retest reliability, convergent validity and divergent validity, we analyzed discriminant and criterion validity of the HCL-32 total score and subscores, to assess screening efficacy.

## Materials and methods

### Participants

We conducted a retrospective study with data collected at the Neuropsychiatry Unit of the Champalimaud Clinical Center, an outpatient psychiatric clinic, between April 2013 and May 2018. Clinical protocol at admission to the Unit involved the application of a battery of self-report instruments, followed by an interview with a clinical psychologist that included a structured diagnostic interview (see section on ‘Other Instruments’ for details), or a clinical assessment by a psychiatrist. While a psychiatrist saw the majority of patients assessed by the psychologist on the same day or a few days later, a subgroup had been referred for psychological and cognitive assessment only. For research purposes, data was also collected from a separate non-clinical sample, recruited using a non-probabilistic sampling technique. For both samples, only adults, 18 years or older, were eligible. Patients with active medical disease, current substance or alcohol dependence, history or clinical evidence of neurological disorders, dementia, illiteracy, or who otherwise did not understand instructions for the study, were excluded. The clinical status of the non-clinical sample was ascertained through a customized questionnaire about medical history and current medication, with a particular focus on psychiatric or neurological history and medication.

### HCL-32

Hypomania Check-List 32 (11) is a screening instrument for lifetime hypomanic episodes. It consists of 32 questions investigating the presence or absence of a variety of symptoms, including inflated self-esteem, decreased need for sleep, augmented communication or pressure to keep talking, subjective experience of racing thoughts, distractibility, increase in goal-directed social or occupational activities, psychomotor agitation and excessive involvement in pleasurable activities (e.g., shopping, hypersexuality, careless driving). Respondents are requested to focus on a given period of “high mood,” and then to indicate whether specific thoughts, emotions and behaviors were present during this period, including low-threshold symptoms such as “making jokes” or “I am less shy and inhibited.” In addition, the HCL-32 includes 8 severity and functional impact items related to the duration of the episodes and to positive and negative consequences across different areas that are not included in the total score. The total score, reflects the sum of one point for each positive response to the 32 questions investigating specific symptoms. Several studies have performed factor analysis of the original HCL-32 or its many translations (11–13, 15, 16, 30) and identified two subscales: “active/elated” and “risk-taking/irritable.” The “active/elated” subscale included items relate to mood elation and improved thinking, self-confidence and sexual activity. The “risk-taking/irritable” subscale includes symptoms of irritable and impatient mood, anger, and risk taking behavior.

### Other instruments

A self-report clinical questionnaire, used as standard clinical protocol at the Neuropsychiatry Unit, was used to collect sociodemographic data and medical history. The Beck Depression Inventory-II (BDI-II) (31, 32) was used to assess severity of depressive symptoms occurring in the last 15 days, while the State-Trait Anxiety Inventory STAI (Form Y, STAI-Y) (33, 34) measured the severity of anxiety symptoms. STAI-Y (State) assesses a transient anxious emotional state while STAI-Y (Trait) assesses a relatively stable predisposition to anxious posture. In a subset of patients Mini International Neuropsychiatric Interview (MINI) (35) was also applied. This is a brief structured diagnostic interview, based on DSM-IV criteria and comprising 15 modules that allow for the clinical diagnosis of several psychiatric disorders and conditions, namely major depressive disorder, dysthymia, suicide risk, manic and hypomanic episode, panic disorder, agoraphobia, social phobia, generalized anxiety disorder, obsessive-compulsive disorder, post-traumatic stress disorder, alcohol abuse or dependence, psychotic disorders, anorexia nervosa, and bulimia nervosa. For this study, we used the European Portuguese translation by Guterres et al. (30).

### Procedures

Permission to use and adapt the HCL-32 to European Portuguese was granted by a member of the team that developed the original scale (11) (Rolf Adolfsson) and authors of the Brazilian Portuguese version (18) (Ricardo Moreno). A team of Portuguese-English bilingual mental health experts, with European Portuguese as the native language, performed several independent adaptations of the validated Brazilian HCL-32 to European Portuguese. According to comparisons with the original English version, minor adjustments were resolved and the research team reached a consensus version of the adaptation to European Portuguese. As mentioned previously, data collection in the clinical sample was part of the routine clinical protocol of the Champalimaud Clinical Center Neuropsychiatry Unit, with patients completing the BDI-II, STAI, and HCL-32 while waiting for the psychology and/or psychiatry appointment, using a pen-and-paper format. In a subset of the patients assessed in a psychology appointment, the MINI was applied. Our local Ethics Committee granted approval for retrospective analysis of fully anonymous and de-identified data from this patient population. Data from a non-clinical sample was collected exclusively for research purposes, in healthy volunteers for whom BDI-II and HCL-32 were applied. The Champalimaud Foundation Ethics Committee also approved procedures for data collection in this group.

### Data analysis

Data analyses were performed using SPSS version 25.0. Results are presented as mean (M) ± standard deviation (SD). All analyses were two-tailed, with significance considered at *p* < 0.05. Continuous measurements were normally distributed according to analysis of kurtosis, skewness, and comparison between mean and median. We performed independent samples *t*-tests to compare age, education and the scores for BDI-II, STAI, and HCL-32 across groups, and Chi-square (χ^2^) analysis for comparisons of sex. Pearson's correlation coefficient (*r*) was used to assess the relationship between HCL-32 and self-report measures of anxiety and depression. Internal consistency of the European Portuguese HCL-32 was assessed using Cronbach's alpha (α) and, based on the two-factor model found in previous studies (11, 18), a Principal Component Analysis (PCA) with varimax orthogonal rotation was conducted to assess factorial structure. To test temporal stability of HCL-32 in the clinical sample, single measures intra-class correlation coefficient (ICC) was employed. Finally, to estimate a cut-off score for screening of BSDs, we used Receiver Operating Characteristic (ROC) curve analysis, using diagnosis of mania/hypomania by MINI as the reference for diagnosis. ROC curves are obtained by plotting the true positive rate (i.e., sensitivity) in function of the false positive rate (1-specificity), with each point in the curve representing a sensitivity/specificity pair corresponding to a decision threshold. Area under the curve (AUC) of these ROC curves reflects the probability that a randomly chosen individual with BSD had a higher HCL-32 score than a randomly chosen individual without BSD, as defined by MINI. The cut-off score was then chosen according to the ROC curve, as the score that maximized sensitivity and specificity. ROC curves using BSD clinical diagnosis by the psychiatrist as the reference standard diagnosis were also obtained.

## Results

### Sample characteristics

Demographic, clinical and psychometric data of the study samples are summarized in Table 1. At the Champalimaud Clinical Center Neuropsychiatry Unit, 463 patients were eligible and had a valid HCL-32 (i.e., with no missing items) collected at the first psychiatry or psychology appointment. A valid HCL-32 was also collected in 62 healthy controls (HC), comprising the non-clinical sample. Among the clinical sample, 382 had a psychiatry appointment, resulting in a diagnosis of bipolar spectrum disorder (BSD, i.e., BD-I, BD-II, or BD-not otherwise specified; *n* = 66), major depressive disorder or major depressive episode (MDD; *n* = 116), or another psychiatric disorder (OPD; *n* = 144)—these groups were considered for further comparisons, as shown in Table 1. In 56 patients, a psychiatric diagnosis was not defined or diagnostic criteria were not met. Among the 181 patients who completed the MINI, 26 fulfilled diagnosis criteria for BSD, 83 for MDD and 31 for OPD, while 41 did not meet diagnostic criteria (DMC). The non-clinical sample was significantly younger compared to the three psychiatric diagnostic groups, with ages ranging from 18 to 47 years. In the three clinical groups, ages ranged from 18 to 83 years, with slightly older participants in the MDD sample. Predominance of female participants was similar across all the samples (54.5–66.4%). The four samples did not differ in terms of formal years of schooling, with the great majority of the participants (86.5%) with 12 or more years of formal education. As expected, BDI-II total scores and anxiety symptoms were higher in the clinical samples, particularly in the MDD group. Following the same pattern, anxiety trait scores differed in the clinical groups, with OPD having the lowest anxiety trait scores. Table 2 summarizes the main psychiatric comorbidities of the clinical groups.

**Table 1:** Demographic and clinical information of the bipolar spectrum disorders sample (BSD), major depressive disorder (MDD), other psychiatric disorders (OPD) and healthy controls (HC).

	**BSD (*****n*** = **66)**		**MDD (*****n*** = **116)**		**OPD (*****n*** = **144)**		**HC (*****n*** = **62)**		
	**Range**	**Mean ± SD**	**Range**	**Mean ± SD**	**Range**	**Mean ± SD**	**Range**	**Mean ± SD**	***p***
Sex (% male)	45.5%		33.6%		36.8%		38.6%		0.180
Age (years)	20–81	46.0 ± 14.8	18–83	50.6 ± 14.3	18–78	47.4 ± 14.8	18–47	27.9± 6.7	0.041^a^ 0.521^b^ 0.081^c^ <0.0001^d^ <0.0001^e^ <0.0001^f^
Education (years)	6–22	14.9 ± 3.4	4–20	14.3 ±3.5	4–25	14.7 ±3.5	9–19	14.0 ± 2.6	0.216^a^ 0.639^b^ 0.346^c^ 0.094^d^ 0.650^e^ 0.187^f^
BDI	3–53	27.3 ± 13.4	10–56	30.4 ± 9.9	2–53	22.6 ± 11.8	0–22	4.6 ± 4.4	0.088^a^ 0.015^b^ <0.0001^c^ <0.0001^d^ <0.0001^e^ <0.0001^f^
STAI-State	23–78	53.3 ± 12.3	23–80	58.9 ±12.3	20–80	52.6 ± 12.0	–		0.004^a^ 0.696^b^ <0.0001^c^
STAY-Trait	25–78	56.0 ± 12.5	33–78	59.4 ± 9.3	24–79	53.5 ± 11.7	–		0.045^a^ 0.146^b^ <0.0001^c^

Independent samples T-tests were performed for statistical comparison between groups:

a BSD vs. MDD;

b BSD vs. OPD;

c MDD vs. OPD;

d BSD vs. HC;

e MDD vs. HC;

f *OPD vs. HC. SD, Standard Deviation. Chi-square (X^2^) test were performed for sex*.

**Table 2:** Main psychiatric co-morbidities of the clinical samples.

	**BSD (*n* = 66) (%)**	**Depressive disorders (*n* = 116) (%)**	**OPD (*n* = 144) (%)**
BD-I	(28) 42.4	(0) 0.0	(0) 0.0
BD-II	(9) 13.6	(0) 0.0	(0) 0.0
Other BSD	(29) 43.9	(0) 0.0	(0) 0.0
Anxiety disorders	(4) 6.1	(13) 11.2	(67) 46.5
OCD	(3) 4.5	(5) 4.3	(20) 13.8
Other comorbidities	(1) 1.5	(1) 0.9	(3) 2.1

*Diagnosis were established by a psychiatrist after clinical assessment. Number of patients and percentages are displayed respectively. BSD, bipolar spectrum disorders; Depressive Disorders, major depressive disorder and major depressive episode; OPD, other psychiatric disorders (e.g., schizophrenia, anxiety disorders, dysthymia, adjustment disorder); BDI-I, Bipolar type 1; BDI-II, Bipolar type II; Other BSD, non-specified BSD; Anxiety Disorders, generalized anxiety disorder, panic disorder, agorafobia, social anxiety disorder, fobias, unspecified anxiety disorder; OCD, obsessive-compulsive disorder; Other comorbidities, other psychiatric diagnosis*.

### General psychometric properties

To assess the factorial structure of HCL-32, we performed a PCA of the 32 HCL items for the patient population (Table 3). PCA with data from the BSD group alone was not possible due to factor invariance of some HCL-32 items. The PCA yielded 32 factors, the first 8 of which with an Eigenvalue of 1 or more. The Kaiser–Meyer–Olkin measure of sampling adequacy was 0.85, indicating the model's adequacy for the factor analysis, while the Bartlett's test of sphericity was statistically significant (*p* < 0.0001), suggesting the data was factorizable. A 2-factor solution, consistent with the deflection of the scree plot and in accordance with Angst et al. (11), was preferred. While the first factor accounted for 19.4%, and the second factor for 8.1% of the total variance of the HCL-32 items, the remaining factors accounted for 5% or less of the total variance. The first factor (Factor 1) included 26 items relating to the previously described “active/elated” factor, while the second factor (Factor 2) had 9 items corresponding to the “risk-taking/irritable” factor, also identified by Angst et al. (11). Internal consistency for the HCL-32 total score was good, with a Cronbach's α coefficient of 0.86. Inter-item correlations were low, ranging between 0.04 and 0.45, but item-total correlations were all significant, ranging between *r* = 0.20 and *r* = 0.53, *p* < 0.001, for items 32 and 15, respectively. The removal of any of the 32 items resulted in an equivalent or lower Cronbach's α. Internal consistency for the HCL-32 “Active/Elated” and “Risk-Taking/Irritable subscores was also adequate with Cronbach's α coefficients of 0.86 and 0.71, respectively.

**Table 3:** Factor structure of HCL-32 after Principal Component Analysis with orthogonal varimax rotation for all the study samples combined (*n* = 525).

**Item**	**Factor loadings**	
	**Factor 1 “Active/elated”**	**Factor 2 “Risk-taking/irritable”**
HCL-1. I need more sleep	0.32	
HCL-2. I feel more energetic and more active	0.41	
HCL-3. I am more self-confident	0.52	
HCL-4. I enjoy my work more	0.54	
HCL-5. I am more sociable (make more phone calls, go out more)	0.54	
HCL-6. I want to travel and do travel more	0.55	
HCL-7. I tend to drive faster and take more risks when driving	0.37	0.40
HCL-8. I spend more money/too much money	0.32	0.37
HCL-9. I take more risks in my daily life (in my work and/or other activities)	0.51	
HCL-10. I am physically more active (sports, etc.)	0.35	
HCL-11. I plan more activities or projects	0.54	
HCL-12. I have more ideas, I am more creative	0.54	
HCL-13. I am less shy or inhibited	0.46	
HCL-14. I wear more colorful and more extravagant clothes/make-up	0.42	
HCL-15. I want to meet or actually do meet more people	0.57	
HCL-16. I am more interested in sex, and/or have increased sexual desire	0.51	
HCL-17. I am more flirtatious and/or am sexually more active	0.54	
HCL-18. I talk more	0.51	
HCL-19. I think faster	0.55	
HCL-20. I make more jokes or puns when I am talking	0.49	
HCL-21. I am more easily distracted	0.44	
HCL-22. I engage in lots of new things	0.52	
HCL-23. My thoughts jump from topic to topic	0.44	
HCL-24. I do think more quickly and/or more easily	0.54	
HCL-25. I am more impatient and/or get irritable more easily		0.54
HCL-26. I can be exhausting or irritating for others		0.55
HCL-27. I get into more quarrels		0.53
HCL-28. My mood is higher, more optimistic	0.40	
HCL-29. I drink more coffee	0.32	0.44
HCL-30. I smoke more cigarettes		0.48
HCL-31. I drink more alcohol		0.43
HCL-32. I take more drugs		0.43

*Small coefficients with values below 0.3 were excluded*.

Across the patient sample (*n* = 463), the HCL-32 total score showed a moderate positive correlation with depressive symptoms as measured by the BDI-II (*r* = 0.38, *p* < 0.0001), as did the “active/elated” subscore (*r* = 0.40 *p* < 0.0001) but not the “risk-taking/Irritable” subscore. State anxiety symptoms were weakly correlated with the HCL-32 total score (*r* = 0.30, *p* < 0.0001) and “active/elated” subscore (*r* = 0.33, *p* < 0.0001) but not the “risk-taking/irritable” subscore. Anxiety traits followed a similar pattern, correlating moderately only with the HCL-32 total score (*r* = 0.37, *p* < 0.0001) and the “active/elated” subscore (*r* = 0.39, *p* < 0.0001). Given the differences in age across study subsamples we also investigated correlations between age and HCL-32 scores (total and both subscores), but did not find significant associations between age and HCL-32 scores in any of the sample groups (data not shown). Across the three groups, correlations with age were significant but very weak, and with *p*-values that would not survive corrections for multiple comparisons (HCL-32 total score, *r* = 0.15, *p* < 0.05; “active/elated” subscore, *r* = 0.15, *p* < 0.05; “risk-taking/irritable” subscore, *r* = 0.1, *p* < 0.05).

Temporal stability of the scale was analyzed by re-administration of the HCL-32 in a subgroup of 78 patients (13 patients with BSD, 10 patients with MDD and 55 patients with OPD; this patient subsample did not differ significantly from the remaining study participants regarding sex, age, education, as well as baseline BDI-II, STAI and HCL-32 scores—data not shown) at follow-up clinical appointments, for comparisons with the first assessment. Test-retest reliability was found to be moderate for this sample (single measures ICC = 0.69), despite the long average interval between assessments (average interval of 175.2 ± 299.0 days).

### Criterion validity

Table 4 summarizes the mean scores of the Portuguese HCL-32 total score, and the “active/elated” and “risk-taking/irritable” subscores of all four-sample groups. As expected, HCL-32 scores differed significantly between all groups, with the BSD group having the higher scores and the HC group the lowest scores. The HCL-32 “Active/Elated” and HCL-32 “Risk-Taking/Irritable” subscore followed the same pattern, except for the lack of difference in risk-taking/irritability scores between the MDD and the OPD sample.

**Table 4:** Mean and standard deviation of HCL-32 total score, HCL-32 “Active/Elated” subscore and “Risk Taking” subscore in the bipolar spectrum disorders sample (BSD), major depressive disorder (MDD), other psychiatric disorders (OPD), and healthy controls (HC).

	**BSD (*n* = 66)**	**MDD (*n* = 116)**	**OPD (*n* = 144)**	**HC (*n* = 62)**	***p***
HCL-32 Total Score	23.0 ± 4.9	19.9 ± 4.0	17.6 ± 5.6	14.6 ± 5.9	<0.0001^a^ <0.0001^b^ <0.0001^c^ <0.0001^d^ <0.0001^e^ 0.001^f^
HCL-32 “Active/Elated” subscore	20.7 ± 3.8	19.0 ± 3.8	16.6 ± 5.3	13.8 ± 5.4	0.003^a^ <0.0001^b^ <0.0001^c^ <0.0001^d^ <0.0001^e^ 0.001^f^
HCL-32 “Risk-Taking/Irritable” subscore	3.9 ± 2.5	1.9 ±1.6	1.8 ±1.8	1.2 ± 1.6	<0.0001^a^ <0.0001^b^ 0.614^c^ <0.0001^d^ 0.005^e^ 0.023^f^

Independent T-tests were performed for statistical comparison between groups:

a BSD vs. MDD;

b BSD vs. OPD;

c MDD vs. OPD;

d BSD vs. HC;

e MDD vs. HC;

f *OPD vs. HC*.

**Figure 1:**
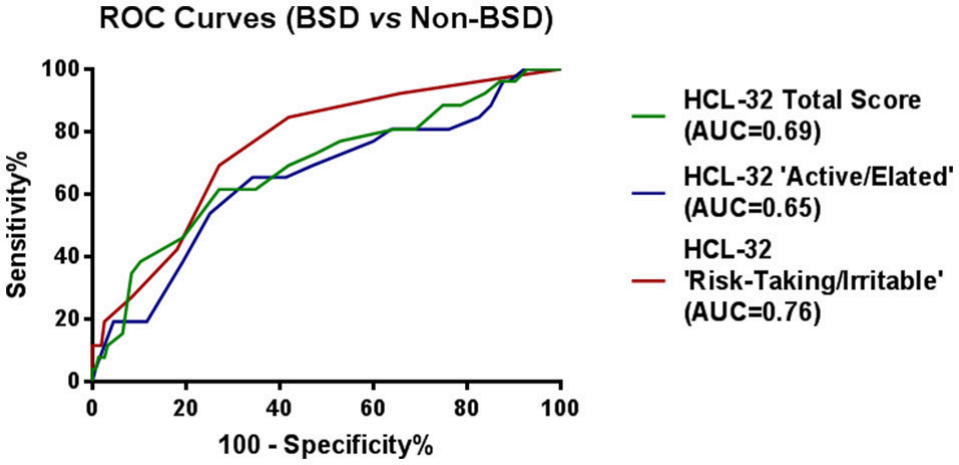
ROC curves showing the power of the HCL-32 scores to discriminate between BSD and MDD, OPD and DMC according to the MINI.

**Figure 2:**
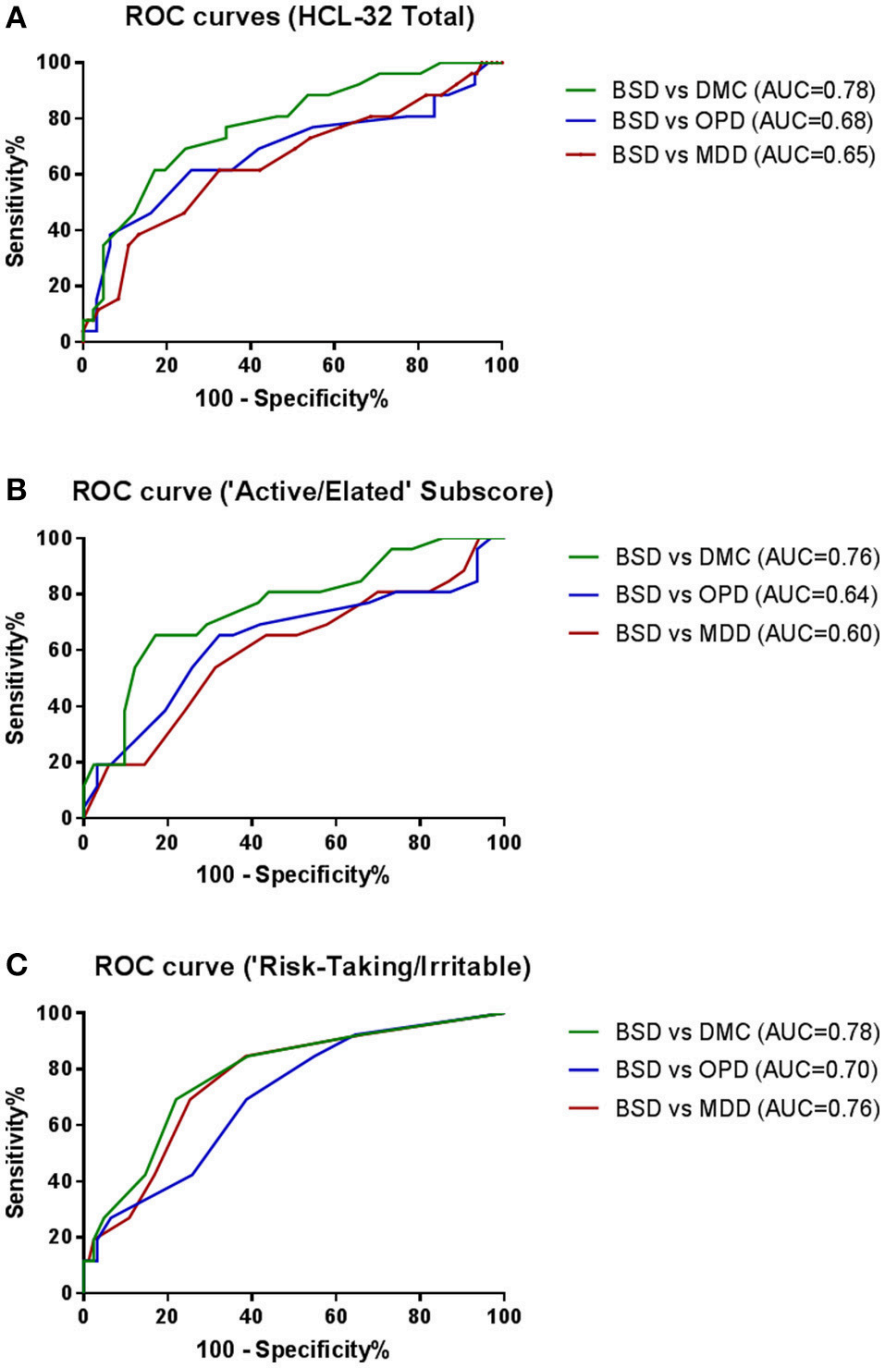
ROC curves showing the ability of the HCL-32 total score **(A)**, the “Active/Elated” subscore **(B)** and the “Risk-Taking/Irritable” subscore **(C)** to discriminate between BSD and MDD, OPD and DMC diagnosis established according to the MINI.

To explore criterion validity of the HCL-32 total score in screening for BPD, we performed a ROC analysis to estimate the optimal cut-off score to discriminate between patients with and without BSD diagnoses, as established by the MINI. The ROC curve in Figure 1, with an area under the curve (AUC) of 0.69, suggests that a cut-off point of 17 yields the best combination of sensitivity (80.7%) and specificity (35.5%) for the HCL-32 total score to distinguish between BSD and non-BSD cases. The ROC curve of the “active/elated” subscale suggested a cut-off score of 14 (sensitivity = 80.8%; specificity = 23.9%; AUC = 0.65). The “risk-taking/irritable” subscale showed particular advantage over the total scores in distinguishing between BSD and non-BSD cases (sensitivity = 84.6%; specificity = 58.1%; AUC = 0.76), with a cut-off score of 2. The same cut-off of 17 for the HCL-32 total score was suggested for ROC curves between patients with BSD and MDD (sensitivity = 80.8%; specificity = 31.3%; AUC = 0.65), OPD (sensitivity = 80.8%; specificity = 22.6%; AUC = 0.68) or DMC (sensitivity = 80.8%; specificity = 53.7%; AUC = 0.78; Figure 2A). The AUC obtained in these subgroup analyses for the HCL-32 subscores ranged from 0.60 to 0.76 for the “active/elated” subscore (Factor 1; Figure 2B) and 0.70 to 0.78 for the “risk-taking/irritable” subscore (Factor 2; Figure 2C). Optimal cut-off scores of the two subscores were also calculated based on the comparison between BSD and non-BSD patients.

**Figure 3:**
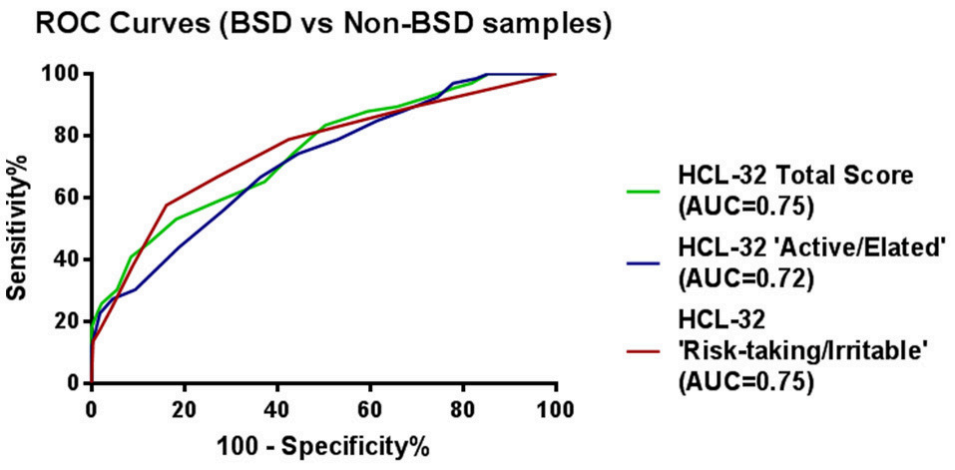
ROC curves showing the power of the HCL-32 scores to discriminate between BSD and non-BSD samples according to a psychiatrist diagnosis.

ROC curve analyses were also performed using psychiatric diagnoses, rather than diagnoses established by the MINI, in order to confirm HCL-32 sensitivity and specificity in a more ecological context. Figure 3 shows that HCL-32 total scale and both of the subscales have similarly good discriminative properties in a more ecological assessment context. Sensitivity and specificity of the cut-off points defined above in comparisons between groups defined according to psychiatric diagnoses are summarized in Table 5.

**Table 5:** Cut-off scores and sensitivity and specificity values for the HCL-32 and its subscores, derived from patients with a MINI interview (left) and those with a psychiatric diagnosis (right).

**MINI**					**Psychiatric diagnosis**				
**BSD** ***vs*****. NON-BSD (MDD**+**OPD**+**CONTROLS)**									
	**Cut-off**	**Sensitivity**	**Specificity**	**AUC**		**Cut-off**	**Sensitivity**	**Specificity**	**AUC**
HCL-32	17	80.7	35.5	0.69	HCL-32	17	89.4	34.2	0.75
FACTOR 1	14	80.8	23.9	0.65	FACTOR 1	14	97.0	22.1	0.72
FACTOR 2	2	84.6	58.1	0.76	FACTOR 2	2	78.8	57.6	0.75
**BSD vs. MDD**									
HCL-32	17	80.8	31.3	0.65	HCL-32	17	89.4	15.5	0.68
FACTOR 1	14	80.8	18.1	0.60	FACTOR 1	14	97.0	8.62	0.63
FACTOR 2	2	84.6	61.5	0.76	FACTOR 2	2	78.8	53.5	0.73
**BSD vs. OPD**									
HCL-32	17	80.8	22.6	0.68	HCL-32	17	89.4	36.2	0.75
FACTOR 1	14	80.8	12.9	0.64	FACTOR 1	14	97.0	25.7	0.73
FACTOR 2	2	84.6	45.2	0.70	FACTOR 2	2	78.8	56.6	0.74
**BSD vs. CONTROLS**									
HCL-32	17	80.8	53.7	0.78	HCL-32	17	89.4	64.5	0.86
FACTOR 1	14	80.8	43.9	0.76	FACTOR 1	14	97.0	38.7	0.85
FACTOR 2	2	84.6	61.0	0.78	FACTOR 2	2	78.8	67.7	0.82

*AUC, Area under the curve*.

## Discussion

In this study we formally adapted the HCL-32 for the Portuguese adult population and assessed its psychometric properties in a first-visit setting, where the intervening clinicians were first establishing a clinical diagnosis, thus providing the best scenario to test the performance of a screening tool. To date, no psychometric instrument for assessment of hypomania has been validated for use among patients who speak European Portuguese. While the scale was found to have adequate psychometric properties, we further demonstrated that the scale is a valid tool to screen adults who have BSD and distinguish them from others, including MDD patients, at admission to an outpatient psychiatry clinic. Similarly to previous research, we also found a dual-factor structure of the scale, namely with “active/elated” and “risk-taking/irritable” subscales. Importantly, we found that the HCL “risk-taking/irritable” subscale may provide advantages for the discrimination between BSD and non-BSD patients.

Regarding the general psychometric properties of the European Portuguese adaptation of the HCL-32, we found that the scale has good internal consistency (Cronbach's α = 0.86), equivalent to that found for the original versions of the scale (0.82 in an Italian sample and 0.86 in a Swedish sample) (11) and higher than the Brazilian Portuguese version of HCL-32 (0.79) (18). Furthermore, a PCA supported a two-factor solution (“active/elated” with 26 items and “risk-taking/irritable” with 9 items) already verified in the original HCL-32 and most other versions (11–13, 15, 16, 36), with only a few studies proposing 3-factor or 4-factor solutions (37), most likely due to sample differences such as size or clinical status. We also performed a test-retest reliability analysis and found it to be adequate, thus supporting the use of the HCL-32 in prospective studies and clinical monitoring. Previous studies reported adequate test–retest reliability over a time interval of 4 weeks (12, 24), whilst in our study, performed in an ecological setting within customary follow-up visits, the time interval was much longer. At the same time, this results could also suggests that HCL-32 is a reliable tool independently on the patient's bipolar disorder stage. Finally, and regarding associations with scores in other instruments, while Forty et al. (38) found no correlation between HCL-32 and BDI-II in a large sample of patients with Bipolar disorder type I (*n* = 230), we found a moderate positive correlation, supporting the presence of pervasive depressive symptoms in BSD patients, possibly because patients tend to seek care during depressive episodes (28). We also found a moderate positive correlation for anxiety symptoms as well as anxiety traits, which is in agreement with Fornaro et al. (39) who found similar correlations in a sample of 280 patients with Major Depressive Episode (STAI-State, *r* = 0.26 and STAI-Trait, *r* = 0.25). Although anxiety disorders are known co-morbidities of BSD (2, 13), our results may also reinforce the proposal that STAI scales don't strictly assess anxiety but also negative affect, resulting in similar correlations with anxiety and depressive symptoms (40).

In addition to symptom severity, the nature of hypomanic symptoms are also related to impairment and prognosis (16), stressing the importance of identifying the structure of the HCL-32. In a Chinese validation of the HCL-32, Wu et al. (13) proposed two subscales based on the same two-factor model found here, that were able to discriminate between patients with MDD, BP-I, and BP-II. The small sample size of our BSD sample did not allow for the assessment of whether these 2 factors could also discriminate between types of Bipolar Disorder.

However, the primary goal of this study was to assess the utility of HCL-32 for screening individuals with BSD, in order to avoid missing a diagnosis of BSD. As expected, total score and subscores of the HCL-32 revealed higher mean hypomania symptom severity scores in the BSD group, followed by the MDD, OPD, and HC groups. Furthermore, we found the scale has good criterion validity to identify patients with BSD, namely when diagnosis was defined formally using the MINI, based on DSM-IV diagnosis criteria. Moreover, criterion validity was conserved when assessed according to psychiatric diagnosis. This finding is particularly interesting, not only because it validates the initial findings based on the MINI, but also because it demonstrates that the scale could be useful as a screening tool in a non-research context. Regarding criterion validity, while most studies proposed a cut-off of 14 (28), in our study a score of 17 was the best cut-off point for the HCL-32 total score to discriminate between BSD patients and the full non-BSD sample, as well as MDD and OPD patients, and HC. In an outpatient clinical screening setting, where it is important to identify high-risk cases for a more comprehensive clinical assessment of BSD, it is preferable to avoid false-negative cases. A cut-off score with high sensitivity may obtain such an effect, while typically at a cost of a lower specificity (14). The cut-off point of 17 for the Portuguese HCL-32 could thus allow for greater accuracy in a two-stage investigation for BSD, as well as potentially reduce unnecessary extensive clinical interviews by correctly identifying patients without BSD. On the other hand, in studies performed in settings in which the proportion of psychiatric disorders is lower than that observed in a clinical sample (i.e., general population, college students, primary care), a higher specificity would be needed (24). Nevertheless, the same cut-off point of 17 performed equally well in distinguishing patients with BSD and healthy volunteers.

Furthermore, when using the HCL-32 subscales, AUC of the ROC curves demonstrated that the two HCL factors (Factor 1: “active/elated,” Factor 2: “risk-taking/irritable”) have differential screening ability to distinguish BSD patients against all samples (MDD, OPD, DMC). Our results suggest that Factor 2 (AUC = 0.76) has better screening ability than both Factor 1 (AUC = 0.65) and the HCL-32 total score (AUC = 0.69). Thus, a shortened 9-item HCL subscale (risk-taking/irritable) may present advantages as a screening tool for BSD relative to both the alternate “active/elated” subscale and the HCL-32 total score. In the original study by Angst et al. (11) these two subscales were similarly defined, but screening benefits were not identified. Soares (18), on the other hand, using the Brazilian Portuguese version of HCL-32, found that factor 2 (“risk-taking/irritable”) rendered good specificity and sensitivity, suggesting the possibility of language or cultural specificities for Portuguese-speaking patients. However, these authors did not propose its use as a subscale due to concerns regarding the presence of irritability and risk taking behaviors in other psychiatric disorders. However, our data for the “risk-taking/irritable” subscale suggests that, with a cut-off point of 2, it performs better not only in the discrimination of BSD and MDD or HC, but also OPD, suggesting that concerns regarding the presence of irritability and risk taking behaviors in other psychiatric disorders may be unwarranted. Furthermore, the advantageous properties of this subscale are mostly reflected in enhanced specificity, rather than sensitivity, also suggesting that false positives may not be a problem for this shortened 9-item HCL subscale. Nevertheless, our sample groups did not include specific groups of psychiatric disorders with a prominent feature of risky behaviors, such as substance abuse, borderline disorder or antisocial disorders, and the items composing factor 2 were administered within the total HCL-32 scale. Thus, further studies regarding the potential use of Factor 2 as a stand-alone screening scale for BSD, namely in clinical populations including patients diagnosed with disorders characterized by risky behaviors, are in need.

Limitations of the present study must also be acknowledged. In fact, the HCL-32 was not validated from the original scale using standard translation and back-translation procedures. However, our adaption of the Brazilian version showed robust psychometric properties that are in complete agreement to previous validation studies and support the use of this version of the HCL-32 in the Portuguese adult population. Another caveat is that the sample size of BSD patients did not allow for detailed subgroup analyses, namely between distinct diagnoses (BD-I, BD-II, etc.) that should be explored in future research. Also, a significant difference in age mean between the control group and the clinical samples may have had an impact in the HCL-32 differences observed in further analyses. However, we did not find significant associations between age and HCL-32 scores in any of the sample groups and significant correlations across the 3 groups were weak and not robust. Furthermore, a previous study (11) described a negative association between age and HCL-32 scores, that was not shown to affect the differences in HCL-32 scores between diagnostic groups. Finally, while data from an ecological context are advantageous with regards to implementing and generalizing findings, there are inherent limitations to retrospective analyses that apply here, for example regarding variability in test-retest reliability testing, and in the definition of a clinical diagnosis by the patient's psychiatrist, in the absence of a formal diagnostic interview.

In conclusion, we have successfully adapted the HCL-32 for use in the Portuguese adult population, and found that it is a useful tool to discriminate between BSD and MDD patients, as well as patients with other psychiatric diagnoses. While a cut-off of 17 was found to have the optimal combination of sensitivity and specificity for the full scale, we also found that a briefer version of HCL, namely the “risk-taking/irritable” subscale, demonstrated adequate, and possibly even enhanced screening properties, supporting potential use of that subscale in a clinical outpatient setting, pending confirmation of these findings. Overall, we thus believe this study makes important contributions to research and clinical activity in BSD in Portugal, while providing support, beyond the Portuguese context, for use of full and reduced versions of the HCL-32 in a real-world outpatient psychiatric clinical setting.

## Author contributions

MC, AM, and AO-M conceived and designed the study. MC, SA, AF, GR, JB-C, JdS, and AO-M collected data. MC, SA, and AM created and organized the study database. MC performed statistical analysis. MC, SA, and AO-M wrote the manuscript that was critically reviewed and approved by the remaining authors.

### Conflict of interest statement

AO-M is funded by a grant from Schuhfried GmBH for norming and validation of cognitive tests. The remaining authors declare that the research was conducted in the absence of any commercial or financial relationships that could be construed as a potential conflict of interest. The handling Editor declared a shared affiliation, though no other collaboration, with one of the authors GR at the time of the review.
